# Diagnostic yield of emergency MRI in non-traumatic headache

**DOI:** 10.1007/s00234-022-03044-2

**Published:** 2022-08-27

**Authors:** Tatu Happonen, Mikko Nyman, Pauli Ylikotila, Harri Merisaari, Kimmo Mattila, Jussi Hirvonen

**Affiliations:** 1 Department of Radiology, Turku University Hospital and University of Turku, Kiinamyllynkatu 4-8, N20520 Turku, Finland; 2 Neurocenter, Turku University Hospital and University of Turku, Turku, Finland; 3 Turku Brain and Mind Center, University of Turku, Turku, Finland; 4 Department of Radiology, Tampere University, Tampere, Finland

**Keywords:** Magnetic resonance imaging, Emergency imaging, Headache, Diagnostic yield

## Abstract

**Purpose:**

Non-traumatic headache is one of the most common neurological complaints in emergency departments. A relatively low diagnostic yield of magnetic resonance imaging (MRI) among outpatients has been previously reported, but studies of emergency patients are lacking. We sought to determine the diagnostic yield of emergency MRI among outpatients presenting to the emergency department with non-traumatic headache.

**Methods:**

In this retrospective cohort study, we analyzed emergency MRI referrals in a tertiary hospital for non-traumatic headache over a five-year period. We recorded patient characteristics, relevant clinical information from the referrals, and imaging outcomes.

**Results:**

In total, 696 emergency patients with non-traumatic headache underwent MRI, most within 24 h of presentation. Significant findings related to headache were found in 136 (20%) patients, and incidental findings in 22% of patients. In a multivariate model, the predisposing factors of the significant findings were age, smoking, nausea, and signs/symptoms of infection. The protective factors were numbness and history of migraine. A predictive clinical score reached only moderate performance.

**Conclusion:**

Although emergency MRI shows headache-related findings in one in five patients, accurate prediction modeling remains a challenge, even with statistically significant predictors and a large sample size.

## Introduction

Non-traumatic headaches are among the most common neurological complaints in emergency departments (ED), reported in ~ 1–4% of patients [1, 2]. They can be classified as either primary or secondary, depending on their etiology [3]. In the emergency setting, various secondary causes of headache can be ruled out by using neuroimaging, which might cause severe neurological morbidity or even death [4].

Major neuroimaging findings among outpatients presenting with non-traumatic headache are rare, and concern < 10% of these patients [5]. Studies using computed tomography (CT) have found secondary causes in 13–15% of emergency patients who had undergone cranial CT for headache, which were mostly intracranial hemorrhages or ischemia [6–8]. Magnetic resonance imaging (MRI) is a suitable alternative, with superior soft tissue characterization and no ionizing radiation, but only a few studies have investigated its yield in an emergency setting [9, 10]. Budweg et al. reported that ~22% (18/82) of their walk-in outpatients had at least potentially significant findings that explained acute headaches. Gilbert et al. found that instead of increasing the prevalence of significant findings, increasing neuroimaging for headaches decreased it [10]. Their results underline the need for support in clinical decision-making regarding the use of imaging to make it more judicious.

Several clinical risk scores have been developed for non-traumatic headache to reduce unnecessary imaging [6, 8, 9, 11]. In these studies, the most frequently presented predictors of intracranial pathology have been age > 50 years, focal neurological deficit, nausea/vomiting, and altered mental status. However, most of these prediction models have been developed for cranial CT. Budweg et al. presented a clinical score for MRI, but so far it has not been validated in a prospective study setting [9].

The aims of this study were to explore emergency MRI findings regarding non-traumatic headache in outpatients presenting to the ED, and to describe these findings in terms of clinical significance. We also aimed to demonstrate factors related to significant imaging outcomes to aid clinical decision-making in the emergency setting.

## Materials and methods

This retrospective cohort study was conducted at Turku University Hospital, an academic tertiary care referral center with an approximate patient catchment area of 480 000. It constitutes the third largest hospital district in Finland. During the study period, the emergency radiology department had a Philips Ingenia 3 Tesla system dedicated to emergency imaging only [12, 13].

We obtained permission from the hospital district board for this study, and patient consent was waived due to its retrospective nature. We first identified 8 772 unique emergency MRI scans conducted between 4/2014 and 1/2019 from picture archiving and communication systems (PACS) and radiological information systems (RIS) using standard MRI codes. The MRI protocols varied, but most included routine sequences such as T1- and T2-weighted imaging, fluid-attenuated inversion recovery (FLAIR), diffusion-weighted imaging (DWI), susceptibility-weighted imaging (SWI), 3D time-of-flight (TOF) arterial angiography, and contrast-enhanced (CE) MRV (selected patients). Imaging data were cross-referenced with those from electronic medical records (EMR).

To identify cases with non-traumatic headache, we first queried referrals with the word “headache.” This search identified 1 862 cases. We excluded already hospitalized inpatients, postoperative patients, patients with a ventriculoperitoneal shunt and patients with a recent head injury. We included all emergency outpatients with non-traumatic headache, regardless of whether the headache was the main symptom, as the proportional significance of headache among all symptoms would be difficult to evaluate retrospectively in these emergency patients. A total of 696 patient cases were included in this study. From the referrals, we recorded the patients’ demographic characteristics, medical history, and other meaningful clinical features mentioned (other symptoms and duration of headache before referral for MRI). Imaging findings were recorded from the MRI reports. Scans with no new findings or notable progression in brain diseases were considered normal. Final diagnoses were then collected from the EMR.

Three board-certified physicians (two fellowship-trained neuroradiologists and a neurologist) reviewed all the referrals and reports, and independently classified findings into *likely explaining headache*, *possibly explaining headache*, *incidental findings with clinical significance*, *incidental findings with no clinical significance*, and *normal*. At least two out of the three study physicians agreed upon 100% of the *likely explaining* and 73% of the *possibly explaining* findings. Similar agreement was reached in 95–99% of *incidental* and *normal* findings. The first two classes were then combined into *findings related to headache*, as they all represented secondary causes of headache. Within this classification (*findings related to headache*, *incidental findings*, *normal scans*), at least two out of the three study physicians agreed upon 100% of all findings. Disagreements were resolved using consensus discussions. The main types of findings were also tabulated (e.g., infarction, hemorrhage, demyelination, arachnoid cyst).

Results are typically expressed as percentages, medians, interquartile ranges (IQR), or odds ratios (OR) with 95% confidence intervals (CI). The normality assumptions were evaluated both visually and using *Saphiro Wilk’s* test. We used the *Chi-squared* test to compare nominal data and the *Wilcoxon rank sum* test as a non-parametric test to compare continuous variables. *P*-values less than 0.25 in univariate analyses were considered sufficiently statistically significant for inclusion in logistic regression [14]. Optimal cut-off points for continuous variables were determined using the *Kolmogorov–Smirnov* metric. A clinical prediction score was derived by multiplying the OR of the predisposing factors and 1/OR of the protective factors by two, and then rounding to the nearest integer [15]. For protective factors, points were assigned if the factor was absent. Receiver operating characteristic (ROC) and area under the curve (AUC) were used to evaluate the diagnostic ability of our model. The optimal cut-off point for the clinical score was determined by *Youden’s J* statistic. In addition to logistic regression, we also evaluated two additional methods to determine whether they would significantly improve the AUC: the Elasticnet (glmnet 3.0.2) model with the Ridge regression model, and the Neural Network (4 hidden layers, neuralnet 1.44.2), the latter with fivefold cross-validation.

The data were analyzed using JMP for Mac (Version 16.1 Pro. SAS Institute Inc., Cary, NC, 1989–2019) and IBM SPSS Statistics for Mac (version 26, copyright IBM Corporation 2019), and R (3.6.3). *P*-values less than 0.05 were considered statistically significant.

## Results

In the total sample of 696 outpatients who presented to the ED with non-traumatic headache and underwent emergency MRI, most were female (*N* = 500, 72%), and their median age was 31 (IQR 23–44) years (Table 1). Most underwent MRI within 24 h of presentation to the ED (96%), and others within a week (median 2 days) of presentation. Duration of headache before referring to emergency MRI was recorded from the referrals, which was ≤ 7 days for 75%, and < 2 days for 42% of the patients. Other aspects of the headache, such as the intensity of pain and localization were not recorded, as such information was not consistently available in the emergency referrals.

**Table 1:** Clinical characteristics of emergency patients who underwent MRI for non-traumatic headache

	All patients *N* = 696	Headache-related finding in MRI *N* = 136	No headache-related MRI finding *N* = 560	*P*-value
Sex, *N* (%)				
Male	196 (28)	44 (32)	152 (27)	0.226
Female	500 (72)	92 (68)	408 (73)	
Age [years], median (IQR)				
Total	31 (23–44)	38 (24–53)	30 (23–42)	< 0.001
Male	31 (20–46)	43 (24–60)	29 (20–43)	0.009
Female	32 (24–44)	37 (24–51)	31 (24–42)	0.025
Medical history, ***N*** (%)				
Pregnancy at presentation	32 (4.6)	3 (2.2)	29 (5.2)	0.138
Smoking	42 (6.0)	13 (10)	29 (5.2)	0.054
Obesity	22 (3.2)	8 (5.9)	14 (2.5)	0.043
Diabetes	18 (2.6)	2 (1.5)	16 (2.9)	0.361
Hypertension	59 (8.5)	17 (13)	42 (7.5)	0.060
Hypercholesterolemia	14 (2.0)	3 (2.2)	11 (2.0)	0.857
Coagulopathy	18 (2.6)	6 (4.4)	12 (2.1)	0.135
Cancer	19 (2.7)	5 (3.7)	14 (2.5)	0.450
Migraine in history	125 (18)	13 (10)	112 (20)	0.004
Headache duration [days], median (IQR)				
Total	3 (0–7)	4 (0–8)	2 (0–7)	0.230
Additional symptoms, *N* (%)				
Nausea	178 (26)	49 (36)	129 (23)	0.002
Vomiting	77 (11)	23 (17)	54 (10)	0.015
Vertigo	153 (22)	31 (23)	122 (22)	0.799
Numbness	218 (31)	27 (20)	191 (34)	0.001
Photophobia	37 (5.3)	7 (5.2)	30 (5.4)	0.922
Visual impairment	211 (30)	46 (34)	165 (29)	0.321
Dysphasia	96 (14)	17 (13)	79 (14)	0.626
Syncope	17 (2.4)	3 (2.2)	14 (2.5)	0.842
Seizure	16 (2.3)	2 (1.5)	14 (2.5)	0.472
Signs/symptoms of infection^1^	43 (6.2)	14 (10)	29 (5.2)	0.026
No other symptoms	94 (14)	13 (10)	81 (14)	0.133
Additional information, *N* (%)				
MRI after 24 h of presentation	30 (4.3)	5 (3.7)	25 (4.5)	0.685
Contrast-enhanced MRI	325 (47)	73 (54)	252 (45)	0.069
Recent head CT for same indication	111 (16)	35 (26)	76 (14)	< 0.001

*IQR*, interquartile range; *MRI*, magnetic resonance imaging; *CT*, computed tomography

*P*-values are associated with *Chi-squared* test for categorical variables, and with *Wilcoxon rank sum* test for continuous variables

^1^Fever, cough, sore throat, runny or stuffy nose, elevated C-reactive protein levels, or neutrophilia

**Fig. 1:**
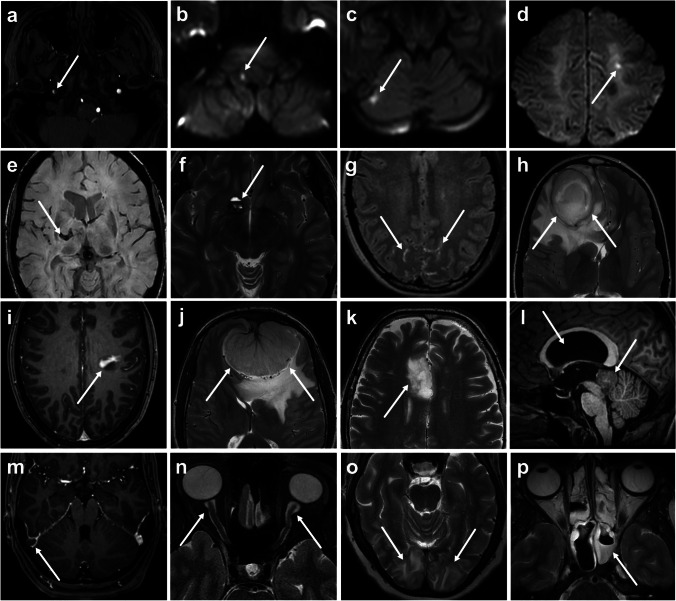
Examples of various emergency MRI findings of pathologies considered significantly related to headache. Examples are: internal carotid artery dissection (**a**), small infarcts (**b**–**d**), intracerebral hemorrhage (**e**), cavernoma (**f**), meningitis (**g**), abscess (**h**), demyelination (**i**), meningioma (**j**), glioma (**k**), central neurocytoma with hydrocephalus (**l**), dural venous sinus thrombosis (**m**), idiopathic intracranial hypertension (**n**), posterior reversible encephalopathy syndrome (**o**), and sphenoid sinusitis (**p**). White arrows denote relevant findings

In total, 136 (20%) patients had a significant headache-related finding in emergency MRI (Fig. 1, Table 2). Among these, most were due to cerebrovascular disease (*N* = 54, 40%), followed by infection/inflammation (*N* = 39, 29%). The most common significant findings were infarction, sinusitis, central nervous system infection, or intracranial tumor. Some less common conditions included mastoiditis, intracranial hyper- and hypotension, Chiari 1 malformation, and posterior reversible encephalopathy syndrome (PRES). Incidental findings with varying clinical significance were found in 154 (22%) scans; mostly white matter lesions, vascular abnormalities, and sinonasal mucosal thickening. Of all the cases, 58% were completely normal.

**Table 2:** Emergency MRI findings in patients imaged for non-traumatic headache

Finding	*N* (%*)
Cerebrovascular disease	**63 (86)**
Infarction	31 (97)
Intracranial hemorrhage	8 (100)
Cerebral venous thrombosis	8 (100)
Arterial dissection/occlusion	7 (100)
Aneurysm	6 (0)
Internal carotid artery stenosis	3 (33)
Infection/inflammation	**41 (95)**
Sinusitis	26 (92)
Central nervous system infection	13 (100)
Mastoiditis	1 (100)
Neuritis	1 (100)
Other	**186 (23)**
Non-specific white matter hyperintensities	52 (0)
Sinonasal mucosal thickening	32 (0)
Intracerebral/meningeal tumor	18 (83)
Leukoaraiosis	14 (0)
Signs of intracranial hypertension	12 (100)
Developmental venous anomaly	9 (0)
Demyelination	8 (75)
Cavernoma	6 (17)
Arachnoid cyst	5 (0)
Benign cyst	4 (0)
Pineal cyst	4 (0)
Hemosiderosis	4 (0)
Mega cisterna magna	4 (0)
Chiari malformation type 1	3 (100)
Signs of intracranial hypotension	3 (100)
Posterior reversible encephalopathy syndrome	2 (100)
Tonsillar ectopy	2 (0)
Pineal cyst apoplexy	1 (100)
Lymphadenopathy	1 (0)
Gliosis	1 (0)
Petrous apex effusion	1 (0)
Total	**290 (47)**

^*^% of findings related to headache and thus considered clinically significant

Among the factors predicting presence of headache-related findings in MRI, age, obesity, history of migraine, nausea, vomiting, numbness, and signs/symptoms of infection reached statistical significance (*P* < 0.05) in a univariate analysis (Table 1). In a multivariate analysis, age, smoking, signs/symptoms of infection, nausea, numbness, and history of migraine remained statistically significant (*P* < 0.05) (Table 3). We found that age over 40 years, smoking, signs/symptoms of infection, and nausea increased the risk of a headache-related finding in emergency MRI, whereas numbness and history of migraine were perceived as protective factors, reducing the risk of such findings. This model had 0.696 ROC AUC, and it correctly classified 81% of the patients. However, classification was correct in 99.8% of patients without headache-related findings, and in only 1.5% of patients with such findings. Neither of the two additionally evaluated models (with Elasticnet and Neural Network), neither provided statistically significant improvement to the AUC.

**Table 3:** Multivariate analysis of predisposing and protective factors for headache-related findings in emergency MRI

	OR	95% CI (lower–upper)	***P***-value
Age over 40 years*	2.6	1.7–3.8	< 0.001
Smoking	2.4	1.1–4.9	0.026
Signs/symptoms of infection	2.3	1.1–4.7	0.025
Nausea	1.9	1.2–2.9	0.004
Numbness	0.6	0.38–0.97	0.034
Migraine in history	0.5	0.26–0.90	0.015

*OR*, odds ratio; *CI*, confidence interval

^*^Age cut-off point determined by *Kolmogorov–Smirnov* metric

**Fig. 2.**
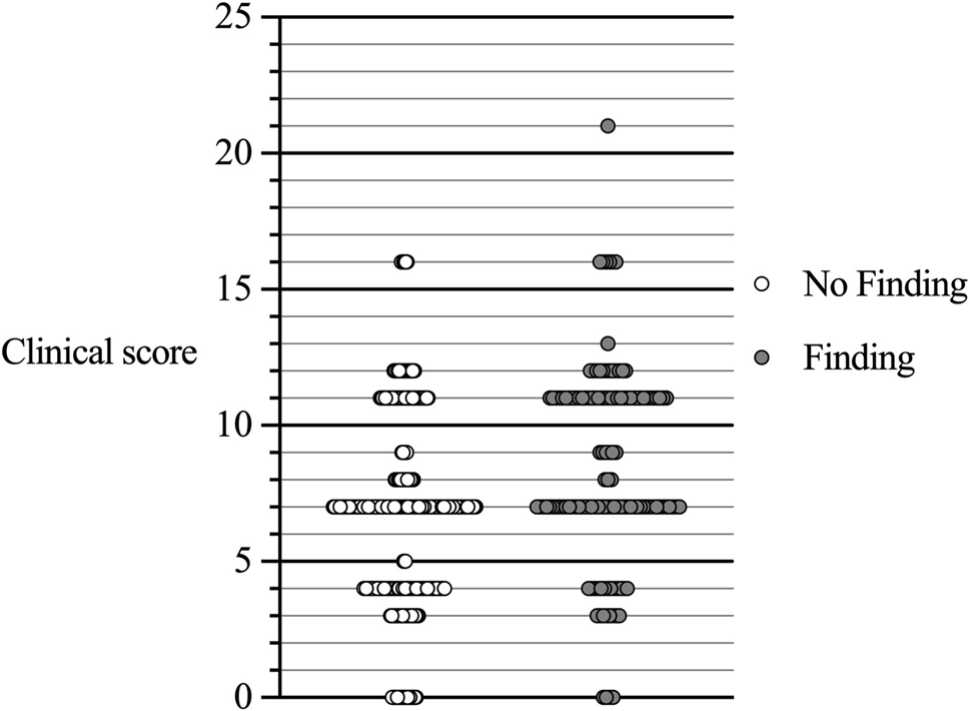
Distributions of the clinical score points within groups with (gray dots) and without headache-related findings (open dots) in emergency MRI. The score consisted of age > 40 years (5 points), smoking (5 p.), signs/symptoms of infection (5 p.) and nausea (4 p.), no numbness (3 p.), and no history of migraine (4 p.).

We then derived the following clinical score to predict headache-related MRI findings: age > 40 years (5 points), smoking (5 p.), signs/symptoms of infection (5 p.), nausea (4 p.), no numbness (3 p.) and no history of migraine (4 p.). The ROC AUC for this model with a single cut-off point of 9 points was 0.625, with a sensitivity of 46% and a specificity of 79%. The clinical score points were considerably scattered in both groups (Fig. 2).

Among the 136 patients with significant findings on MRI, 35 patients (26%) had previous CT scans, of which 29% were unremarkable. For example, all previous CT scans for patients with acute infarction on MRI (*N* = 10) were normal).

We were not able to reliably evaluate whether headache was the primary presenting symptom in all patients because many had several symptoms. However, the 94 (14%) patients with headache as the only (and thereby primary) presenting symptom had similar rates of significant pathology on MRI, although they were more likely to be younger, female, pregnant, and with a history of migraine. In addition, they had a longer duration of headache before imaging than patients with additional symptoms.

Regarding the diagnoses of ED discharge/headache etiologies, 25% were diagnosed with a primary headache syndrome, mostly migraine or tension-type headache (Table 4). Thirty percent had a secondary cause of headache (either new or chronic), and the remaining 45% were discharged with a diagnosis of “non-specified headache” due to a lack of further knowledge on the etiology of the headache.

**Table 4:** Diagnoses at emergency department discharge/headache etiologies

Diagnosis/headache etiology	*N* (%)
Primary headache syndromes	**176 (25)**
Migraine	140 (20)
Tension-type headache	35 (5.0)
Cluster headache syndrome	1 (0.1)
Secondary headache syndromes	**205 (30)**
Cerebrovascular disease	68 (9.8)
Cerebral infarction	33 (4.7)
Transient ischemic attack	11 (1.6)
Arterial occlusion/stenosis	9 (1.3)
Cerebral venous thrombosis	8 (1.1)
Intracranial hemorrhage	7 (1.0)
Infectious diseases	42 (6.0)
Meningitis/encephalitis	23 (3.3)
Sinusitis	9 (1.3)
Ocular etiology	29 (4.2)
Neoplasms	14 (2.0)
Idiopathic intracranial hypertension	13 (1.9)
Demyelinating diseases	7 (1.0)
Intracranial hypotension/post-lumbar puncture headache	3 (0.4)
* Other:* anemia, asidosis, mental disorders, epilepsy, cerebral aneurysm, Bell’s palsy, drug-induced headache, pregnancy-induced headache, hydrocephalus, and Arnold-Chiari syndrome	29 (4.2)
Unknown etiologies	**315 (45)**
Non-specified headache	315 (45)

Note that discharge diagnoses may include chronic diseases, and not only those found on emergency MRI

## Discussion

In this large-scale study of emergency outpatients, we found that the majority who underwent emergency MRI for non-traumatic headache had normal scans, whereas about 20% had significant findings that potentially explained the headache. Thus, about five patients needed to be scanned to diagnose one patient with significant intracranial pathology. Although we found significant predisposing and protective factors, the performance predictive model was only moderate, and the model could not accurately detect patients with headache-related findings. Judicious use of emergency neuroimaging to rule out secondary causes of non-traumatic headache remains a challenge, even using MRI.

Regarding MRI findings in patients with headache in general, a fairly recent meta-analysis by Jang et al. [5] found potentially significant abnormalities assessed by MRI in 5.7% (95% CI: 1.6–20%) of all patients suspected of primary headache. Budweg et al. found that ~22% (18/82) of their walk-in patients had findings that could at least potentially explain their acute headache, of whom 10% (8 patients) had findings that were considered significant [9]. In both studies, only patients with a provisional diagnosis of a primary headache were included. Our data showed that the yield of MRI was higher among emergency patients (20% with significant findings), most likely due to the higher prevalence of severe intracranial acute-onset pathology (e.g., intracranial hemorrhage, infarctions, and central nervous system infections). We also decided to cover all emergency outpatients who had non-traumatic headache, including patients with abnormal neurological findings and suspicion of high-risk pathology. Our patients were imaged with significantly shorter latency (96% within 24 h) than that in the previous study by Budweg et al.: 72% of their patients had MRI within three days of presentation, and 54% were imaged on the same day.

Our most common headache-related findings were similar to those reported by Budweg et al.; in both studies, findings such as signs of intracranial hypertension, meningitis, and cerebral infarction were prevalent. Moreover, our data showed various, less common causes that were not met in the previous smaller sample, including Chiari 1 malformation, arterial dissection and occlusion, PRES and signs of intracranial hypotension. When compared to the previous studies using CT for acute headaches, they reported similar prevalences of cerebrovascular conditions (intracranial hemorrhages and ischemia) and newly detected neoplasms, but a lower prevalence of conditions that are more identifiable by MRI (such as infectious diseases and intracranial hypertension) [6–8]. We found recent infarcts (identified with DWI) in 30 patients (4% of all, 22% of those with significant findings). Most of these infarcts were small and often punctate. None of the patients had motor loss, and the prevalence of numbness was not higher than among other patients with significant findings. These small infarcts thus were unlikely to cause major neurological deficits, which are usually primarily imaged with CT anyway. In fact, a third of these patients had previous CT scans, all with unremarkable findings.

The proportion of incidental findings discovered was similar to that of the significant findings, and also to that reported for patients with a new primary headache [16]. Kim et al. reported incidental abnormalities in 25% of new primary headache patents scanned with MRI, of which white matter hyperintensities and sinonasal abnormalities not related to headache were the most common. Our findings were similar, confirming the high prevalence of incidental findings and similarities between emergency and non-emergency settings. Even clinically insignificant incidental findings may cause unnecessary worry in patients and healthcare providers.

We found that age > 40 years, smoking, signs/symptoms of infection, and nausea significantly increased the risk of abnormal headache-related findings in MRI, whereas numbness and history of migraine reduced this same risk. Of these factors, older age and nausea were the only ones reported in the previous CT and MRI scores [6, 8, 9, 11]. A focal neurological deficit was reported as a major risk factor in every CT score, but this was not the case in our data. One explanation is that such patients may have undergone a CT instead of MRI. None of the previous studies reported factors that would reduce the risk of significant findings. The reason why known migraine was perceived as a protective factor may be that a new type of headache in a migraine patient could still be migraine rather than due to a secondary cause. Among the patients with a history of migraine, only 10% had meaningful findings in MRI.

Our model predicting significant imaging outcomes among emergency patients provided limited value with low sensitivity and moderate specificity. The clinical score in the model of Budweg et al. had considerably higher sensitivity (100% vs. 46%), similar specificity (82% vs. 79%), and a superior ROC AUC (0.94 vs. 0.63). One reason for these differences may be that their model was developed for patients in an outpatient walk-in clinic setting, which presents a narrower spectrum of imaging outcomes and symptoms than that among emergency patients.

According to our model, a typical patient who is the least likely to show abnormal findings is a young non-smoking patient with a history of migraine. Our multivariate model could not accurately detect patients with headache-related findings. The moderate performance of our model reflects how difficult it is to create accurate, universal risk scores for clinical use in a heterogeneous patient population with various symptoms, risk factors, and imaging findings.

One of the major strengths of this study is the routine use of MRI in the emergency radiology department and a large sample size. In addition, this study represents a true clinical situation and offers a real-world overview of emergency patients with non-traumatic headache. We utilized a data-driven approach by querying the referrals for specific symptoms, instead of relying on diagnosis codes.

Our study is limited by its retrospective design. All the relevant data may not have been available from the emergency referrals. Some referrals may have been incomplete or imprecise, and therefore the true prevalence of risk factors may have been underestimated. In addition to specific symptoms, relevant comorbidities and medical history may have been missing. Lack of relevant data may have contributed to the performance of the predictive model. Regarding symptoms, a potential limitation of our study is that we could not reliably record from the referral data whether headache was the primary presenting symptom. However, the rate of significant findings was not significantly different between patients with only headache and those with additional symptoms (Table 1), suggesting that our results are not significantly biased because of this limitation. A prospective confirmation of current findings in the future is warranted before claims of clinical utility can be made.

Our results are only applicable to emergency MRI, which may not be suitable or readily available in all institutions, whereas CT is usually the method of choice in acute neuroimaging of headache patients. We focused on the first-line use of emergency MRI and did not include headache patients undergoing CT only. Regarding generalizability, our study is limited by the fact that we did not include headache patients not scheduled for emergency MRI. Therefore, we do not know the factors that contributed to the need for emergency MRI. Our results on the diagnostic yield are only generalizable to patients deemed to require emergency neuroimaging, with the goal of identifying patients in whom imaging is unlikely to yield significant findings. These results provide novel information on the diagnostic yield in this patient group when emergency MRI is readily available and commonly used in the emergency department. Regarding the clinical value of emergency MRI findings, MRI likely altered the clinical management of patients with newly discovered neurological disorders such as cerebrovascular disease (including acute infarction), demyelinating and infectious disease, and idiopathic intracranial hypertension. In addition, even patients with worrisome symptoms who had normal emergency MRI may have been safely discharged.

## Conclusions

In conclusion, we found that the majority of emergency patients with non-traumatic headache do not present significant abnormalities in MRI. Even with significant predictors indicating abnormal findings, predictive modeling to promote using neuroimaging judiciously remains a challenge. Larger populations with complete clinical characterization may be needed to create more accurate predictive models for emergency MRI.

## Data Availability

Data cannot be publicly shared because of the national legislature on the patient data. All relevant data is in the manuscript.

## Declarations

### Guarantor

JH
